# VRK3-mediated nuclear localization of HSP70 prevents glutamate excitotoxicity-induced apoptosis and Aβ accumulation via enhancement of ERK phosphatase VHR activity

**DOI:** 10.1038/srep38452

**Published:** 2016-12-12

**Authors:** Haengjin Song, Wanil Kim, Sung-Hoon Kim, Kyong-Tai Kim

**Affiliations:** 1 Department of Life Sciences, Pohang University of Science and Technology, Pohang, Gyeongbuk, 37673, Republic of Korea; 2 Division of Integrative Biosciences and Biotechnology, Pohang University of Science and Technology, Pohang, Gyeongbuk, 37673, Republic of Korea

## Abstract

Most of neurodegenerative disorders are associated with protein aggregation. Glutamate-induced excitotoxicity and persistent extracellular signal-regulated kinase (ERK) activation are also implicated in neurodegenerative diseases. Here, we found that vaccinia-related kinase 3 (VRK3) facilitates nuclear localization of glutamate-induced heat shock protein 70 (HSP70). Nuclear HSP70 leads to enhancement of vaccinia H1-related phosphatase (VHR) activity via protein-protein interaction rather than its molecular chaperone activity, thereby suppressing excessive ERK activation. Moreover, glutamate-induced ERK activation stimulates the expression of HSP70 and VRK3 at the transcriptional level. Downregulation of either VRK3 or HSP70 rendered cells vulnerable to glutamate-induced apoptosis. Overexpression of HSP70 fused to a nuclear localization signal attenuated apoptosis more than HSP70 alone. The importance of nuclear localization of HSP70 in the negative regulation of glutamate-induced ERK activation was further confirmed in VRK3-deficient neurons. Importantly, we showed a positive correlation between levels of VRK3 and HSP70 in the progression of Alzheimer’s and Parkinson’s diseases in humans, and neurons with HSP70 nuclear localization exhibited less Aβ accumulation in brains from patients with Alzheimer’s disease. Therefore, HSP70 and VRK3 could potentially serve as diagnostic and therapeutic targets in neurodegenerative diseases.

Neurodegenerative disorders, such as Alzheimer’s disease (AD), Parkinson’s disease (PD), and Huntington’s disease, are characterized by selective loss of neurons in motor, sensory, or cognitive brain areas. Due to the accumulation of intracellular inclusions or extracellular protein aggregates in specific brain regions, many neurodegenerative disorders are also known as proteinopathies^1^. In AD, β-amyloid (Aβ) plaques and neurofibrillary tangles consisting of aggregated Aβ peptide and hyperphosphorylated tau aggregates, respectively, accumulate in extracellular and intracellular space^2^,^3^. In PD, Lewy bodies containing aggregated α-synuclein are also deposited intracellularly^4^.

Heat shock proteins (HSPs) are the major class of molecular chaperones that interact with and stabilize client proteins to maintain their native conformation, thereby preventing protein misfolding and aggregation under stress conditions^5^. HSPs are induced by various stresses such as heat shock, ischemia, hypoxia, heavy metals, and amino acid analogs^6^,^7^, and many are upregulated in tissue from aged animals and elderly patients with neurodegeneration^8^,^9^. The HSP70 family, one of the most conserved molecular chaperone families, supports the degradation, transport, and dissociation of misfolded protein complexes and prevents aggregation of client proteins^10^,^11^,^12^,^13^. In eukaryotes, HSP70 is found in almost all intracellular compartments, including the cytosol, endoplasmic reticulum, and the mitochondria^14^. The expression of HSP70 is very low under normal physiological conditions, but three members of HSP70 family are induced in response to stress^6^, with heat shock stress particularly inducing their rapid and transient relocation to the nucleus^15^. A recent study further identified a new HSP70 transport pathway mediated by the carrier protein Hikeshi under heat shock stress conditions^16^.

A large body of evidence supports a protective role of HSP70 in humans and animal disease models. For example, in AD, HSP70 inhibits Aβ oligomerization, enhances Aβ clearance, restores tau homeostasis, and suppresses neuronal apoptosis^17^,^18^,^19^. In PD, HSP70 enhances α-synuclein refolding and degradation, thereby decreasing α-synuclein aggregate formation and toxicity^20^. However, beyond its role as a chaperone, the mechanisms by which HSP70 functions in cells and exerts neuroprotection are poorly understood.

Persistent activation of extracellular signal-regulated kinase 1/2 (ERK 1/2)-induced neuronal degeneration is also implicated in neurodegenerative diseases^21^,^22^. ERK 1/2, which belongs to the family of mitogen-activated protein kinases (MAPKs), regulates many cytoplasmic and nuclear targets by phosphorylation, and mediates several important cellular functions, including proliferation, migration, and differentiation^23^,^24^. Although ERK 1/2 activity is generally involved in cell survival, growing evidence suggests that ERK 1/2 also mediates apoptosis depending on the intensity and duration of its activity and subcellular distribution^23^,^24^. The regulation of cell survival and death relies on the balance between pro- versus anti-apoptotic signals transmitted by ERK 1/2^23^,^24^. Sustained activation of ERK 1/2 causes its translocation to the nucleus and can promote neuronal death via transcriptional regulation^25^. The active ERK in the nucleus is regulated by vaccinia H1-related phosphatase (VHR) that specifically dephosphorylates and inactivates nuclear ERK 1/2^26^,^27^.

Glutamate, the major excitatory neurotransmitter in the mammalian central nervous system^28^, has pivotal roles in neuronal development, synaptic transmission, and plasticity that underlie learning and memory, emotion, sensation, and motor function; however, glutamate is also involved in neurological diseases^29^. Excessive activation of glutamate receptors impairs intracellular calcium homeostasis, increases generation of reactive oxygen species, and alters the activation of kinases, including ERK 1/2, and proteases that degrade proteins, membranes, and nucleic acids^30^. This phenomenon, referred to as ‘glutamate neurotoxicity’, results in damage to dendrites and even cell death. Although the exact mechanisms that initiate and contribute to the progression of neurodegeneration remain unknown, neurodegenerative diseases share common pathological features, such as persistent ERK 1/2 activation^21^,^22^, oxidative stress^31^, and excitotoxicity^30^.

In this study, we investigated endogenous protective mechanisms against glutamate-induced neurotoxicity. We found that VRK3 interacts with glutamate-induced HSP70 and promotes its nuclear localization. Nuclear HSP70 enhances VHR phosphatase activity and leads to attenuation of persistent ERK activation and cell death caused by glutamate excitotoxicity. Results from VRK3-deficient neurons further confirmed the role of HSP70 nuclear localization in the regulation of glutamate-induced prolonged ERK activation that triggers cell death. Furthermore, in brains of AD and PD patients, we observed increased levels of VRK3 and HSP70 and the presence of Aβ plaques that were deficient in nuclear HSP70, suggesting that nuclear HSP70 protects cells from protein aggregation-induced neuronal death. Hence, we conclude that VRK3-mediated nuclear localization of HSP70 plays a neuroprotective role by reducing glutamate-induced prolonged ERK activity that causes cell death.

## Results

### HSP70 interactes with and enhances VHR phosphatase activity

**Figure 1:**
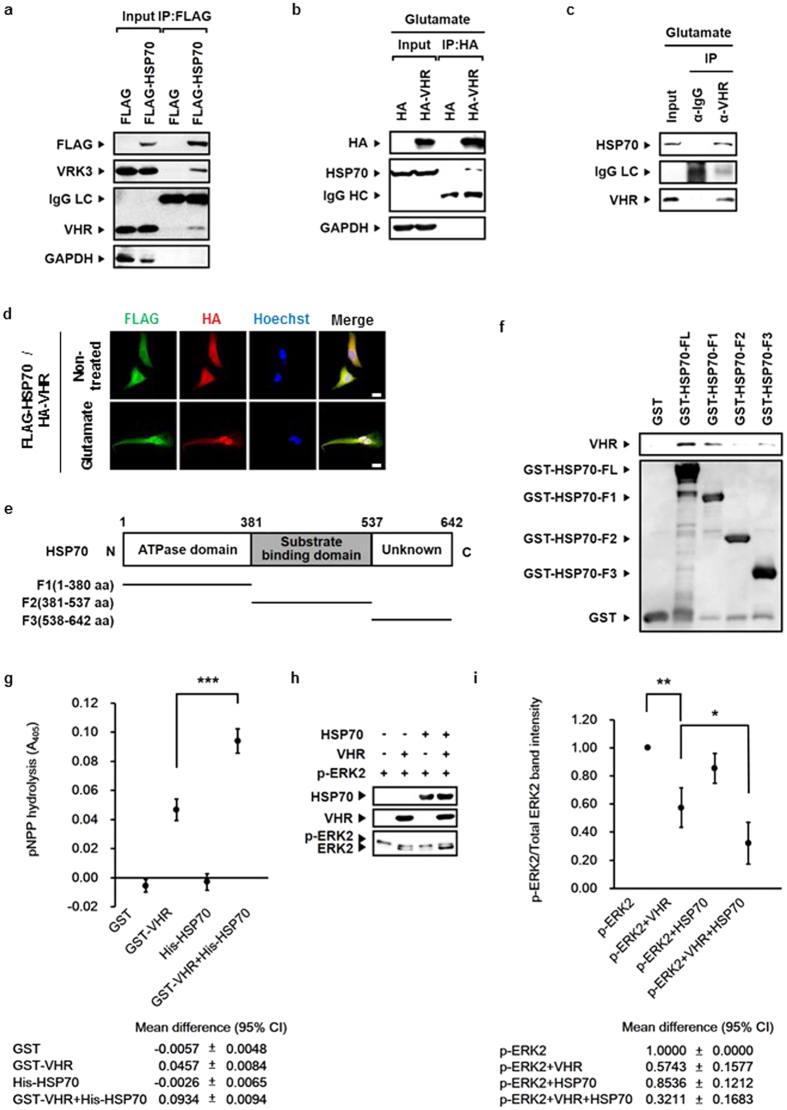
HSP70 interacts with and enhances VHR phosphatase activity. (**a**) Endogenous VHR and VRK3 immunoprecipitated with an antibody against anti-FLAG in SH-SY5Y cell lysates expressing FLAG- HSP70. (**b**) Endogenous HSP70 immunoprecipitated with an antibody against anti-HA in glutamate-treated SH-SY5Y cell lysates expressing HA-VHR. (**c**) Endogenous HSP70 immunoprecipitated with endogenous VHR in glutamate-treated SH-SY5Y cell lysates. (**d**) FLAG-tagged HSP70 and HA-tagged VHR were mainly co-localized in the nucleus of SH-SY5Y cells treated with glutamate. Scale bar, 20 μm. (**e**) Domain structure of HSP70 and schematic representation of HSP70 fragments. (**f**) HSP70 bound to VHR with its ATPase domain and a functionally unknown region (C-terminal region). (**g**) A 2-fold increase in VHR phosphatase activity by HSP70. *In vitro* VHR phosphatase assay was performed using HSP70 with pNPP. Values are shown as mean ± standard deviation (s.d.), n = 3. Student’s t-tests, ***P < 0.001. (**h**) HSP70 enhanced VHR phosphatase activity in a chaperone activity-independent manner. *In vitro* VHR phosphatase assay was performed using HSP70 with recombinant p-ERK2. (**i**) Quantification of the percentage of p-ERK2 versus total ERK2 as in (h). Values are normalized to p-ERK2-only control and shown as mean ± s.d., n = 3. Student’s t-tests, *P < 0.05, **P < 0.01.

We identified VHR and VRK3 as binding partners of HSP70 by performing co-immunoprecipitation assay using SH-SY5Y cell lysates overexpressing the stress-inducible HSP70 form, HSP70A1A (Fig. 1a). VHR has a role in specific dephosphorylation and inactivation of nuclear ERK^26^,^27^, and VRK3 acts as a mediator protein^32^. We first examined the relationship between HSP70 and VHR. Inducible HSP70 immunoprecipitated with an antibody against anti-HA in glutamate-treated SH-SY5Y cells expressing HA-VHR (Fig. 1b). In addition, the interaction of endogenous VHR with inducible HSP70 was further confirmed in glutamate-treated SH-SY5Y cells (Fig. 1c). Glutamate treatment induced nuclear translocation of both FLAG-tagged HSP70 and HA-tagged VHR, proteins that were predominantly localized in the cytoplasm under normal, non-stressed conditions (Fig. 1d). HSP70 and VHR were mainly co-localized in the nucleus of glutamate-treated SH-SY5Y cells. This data indicates that glutamate-induced HSP70 interacts with VHR predominantly in the nucleus.

HSP70 consists of two highly conserved domains: the N-terminal nucleotide binding domain and the C-terminal substrate binding domain^33^. To clarify whether HSP70 directly interacts with VHR, and to identify the specific VHR-binding region in HSP70, we conducted the GST pull down assay using GST-tagged full-length HSP70 and its fragments (F1, amino acids 1-380; F2, amino acids 381-537; F3, amino acids 538-642; Fig. 1e). GST-HSP70, but not GST control, specifically associated with VHR (Fig. 1f). Also, HSP70 F1 and F3 fragments, but not the F2 fragment, bound to VHR. To determine whether the binding of HSP70 affects VHR activity, we performed *in vitro* phosphatase assay (Fig. 1g–i). Compared with VHR alone, the addition of HSP70 induced a 2-fold increase in VHR phosphatase activity when either p-nitrophenyl phosphate (pNPP; Fig. 1g) or purified recombinant p-ERK2 protein (Fig. 1h,i) was used as a substrate. These results indicate that HSP70 enhances VHR phosphatase activity.

### VRK3 promotes nuclear localization of HSP70 induced by glutamate

**Figure 2:**
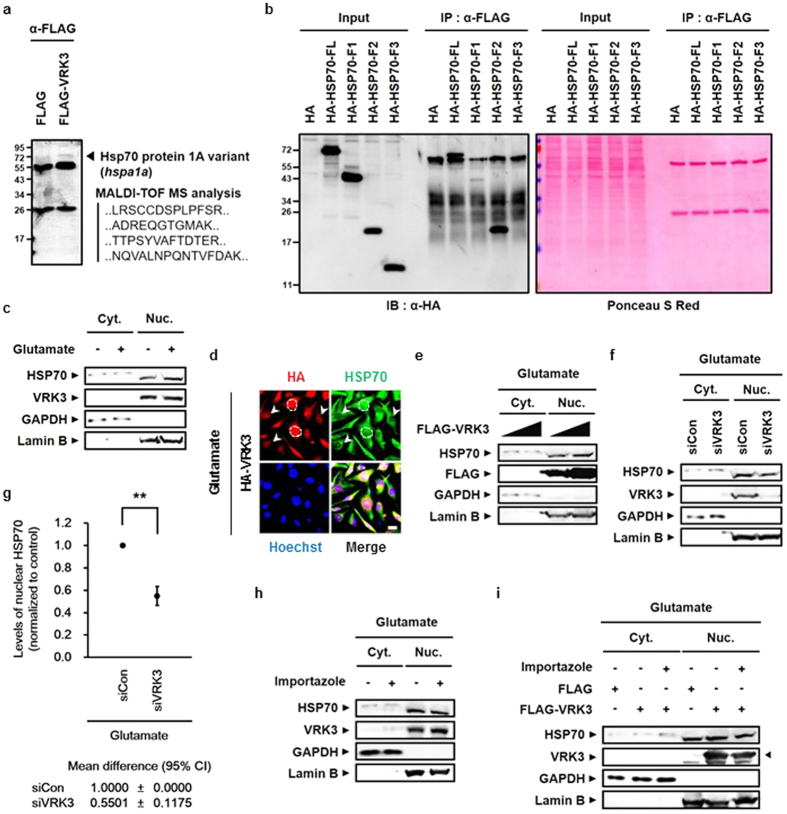
VRK3 promotes nuclear localization of HSP70 in glutamate-treated SH-SY5Y cells. (**a**) HSP70 bound to FLAG-tagged VRK3 in HEK2FT cells. (**b**) FLAG-tagged VRK3 bound to the HA-tagged substrate binding domain of HSP70 with high affinity. (**c**) Nuclear localization of endogenous HSP70 was increased in glutamate-treated SH-SY5Y cells. (**d**) HA-tagged VRK3 and endogenous HSP70 were co-localized in glutamate-treated SH-SY5Y cells. The dotted lines represent the boundary between nucleus and cytoplasm; White arrowheads highlight cells expressed relatively low level of HA-VRK3. Scale bar, 20 μm. (**e**) Overexpression of FLAG-tagged VRK3 facilitated nuclear localization of HSP70 in dose-dependent manner. (**f**) Inhibition of glutamate-induced nuclear localization of HSP70 in cells with VRK3 knockdown. (**g**) Quantification of the levels of nuclear HSP70 as in (**f**). Values are normalized to control and shown as mean ± s.d., n = 3. Student’s t-tests, **P < 0.01. (**h**) Glutamate-induced nuclear localization of HSP70 was suppressed by pre-treatment with importazole, a nuclear transport receptor importin-β inhibitor. (**i**) Nuclear localization of HSP70 facilitated by FLAG-tagged VRK3 was also suppressed by importazole treatment.

We next examined the relationship between HSP70 and VRK3. The interaction of these two proteins confirmed by mass spectrometry analysis on FLAG-VRK3- expressing 293FT cell lysates immunoprecipitated with an antibody against anti-FLAG (Fig. 2a). To identify the VRK3-binding region in HSP70, we expressed HA-tagged full length HSP70 or its fragments along with FLAG-VRK3 in SH-SY5Y cells (Fig. 2b). Although it has a different electrophoretic mobility than that seen in the input, HA-tagged full length HSP70 retrieved in the immunoprecipitation. The HSP70 F2 fragment, which contains the substrate binding domain, was found to interact with VRK3 (Fig. 2b). For the interaction to occur, nuclear translocation of HSP70 is necessary, because VRK3 is mainly localized in the nucleus due to its nuclear localization signal (NLS)^34^. HSPs are induced by various stresses^6^,^7^ and especially, in response to heat shock stress, HSP70 are rapidly expressed and transiently relocated from cytoplasm into the nucleus^6^. To verify whether treatment with excessive glutamate induces nuclear translocation of HSP70, we tested cytoplasmic and nucleoplasmic fractions of non-treated and glutamate-treated SH-SY5Y cells (Fig. 2c). Nuclear HSP70 was markedly increased in cells treated with glutamate compared with control. In addition, co-localization of HA-tagged VRK3 and endogenous HSP70 was detected in glutamate-treated SH-SY5Y cells (Fig. 2d). The amount of nuclear HSP70 was increased in cells overexpressing VRK3. To clarify whether VRK3 binding affects nuclear localization of HSP70, we analyzed cytoplasmic and nucleoplasmic fractions of glutamate-treated SH-SY5Y cells (Fig. 2e, Supplementary Fig. S1). VRK3 promoted nuclear localization of glutamate-induced HSP70 in dose-dependent manner (Fig. 2e), compared with control which showed no differences in levels of nuclear HSP70 between the groups (Supplementary Fig. S1). In contrast, cells reducing VRK3 expression using siRNAs showed markedly suppressed nuclear localization of glutamate-induced HSP70 (Fig. 2f,g). We also tested the effects of overexpression or knockdown of VHR on nuclear localization of HSP70 in glutamate-treated SH-SY5Y cells (Supplementary Fig. S2a,b). There were no significant differences in levels of nuclear HSP70 between the groups. Together, these findings reveal that VRK3 facilitates glutamate-induced nuclear localization of HSP70.

Regulation of the nucleocytoplasmic protein transport is mediated by members of the importin β family^35^. Importin β family carriers interact with their specific cargos and facilitates transfer to the nucleus through the nuclear pore complex (NPC) upon binding^35^. To elucidate the mechanism of enhanced nuclear localization of HSP70 by VRK3, we pre-treated cells with importazole, an inhibitor of importin-β transport receptors, before glutamate treatment and then analyzed cytoplasmic and nucleoplasmic fractions of SH-SY5Y cells (Fig. 2h,i). Pre-treatment of the cells with importazole resulted in suppression of glutamate-induced HSP70 nuclear translocation, even though the expression of endogenous VRK3 was increased (Fig. 2h). Consistent with previous results, VRK3 overexpression promoted nuclear localization of HSP70, however, pre-treatment with importazole resulting in reduced nuclear HSP70 (Fig. 2i). Taken together, this data indicates that VRK3 supports nuclear retention of glutamate-induced HSP70 rather than assisting in its translocation to the nucleus.

### Glutamate-induced active ERK stimulates the gene expression of both HSP70 and VRK3, and inhibits its sustained activity that triggers cell death

Although ERK is associated with cell survival, it is also linked to cell death. ERK 1/2 determines cell fate based on its abundance, duration of activation, and subcellular distribution^23^,^24^. Several studies have focused on the detrimental role of persistent ERK activation under stress^36^,^37^,^38^. Furthermore, prolonged ERK activation is implicated in glutamate-induced cell death^37^.

**Figure 3:**
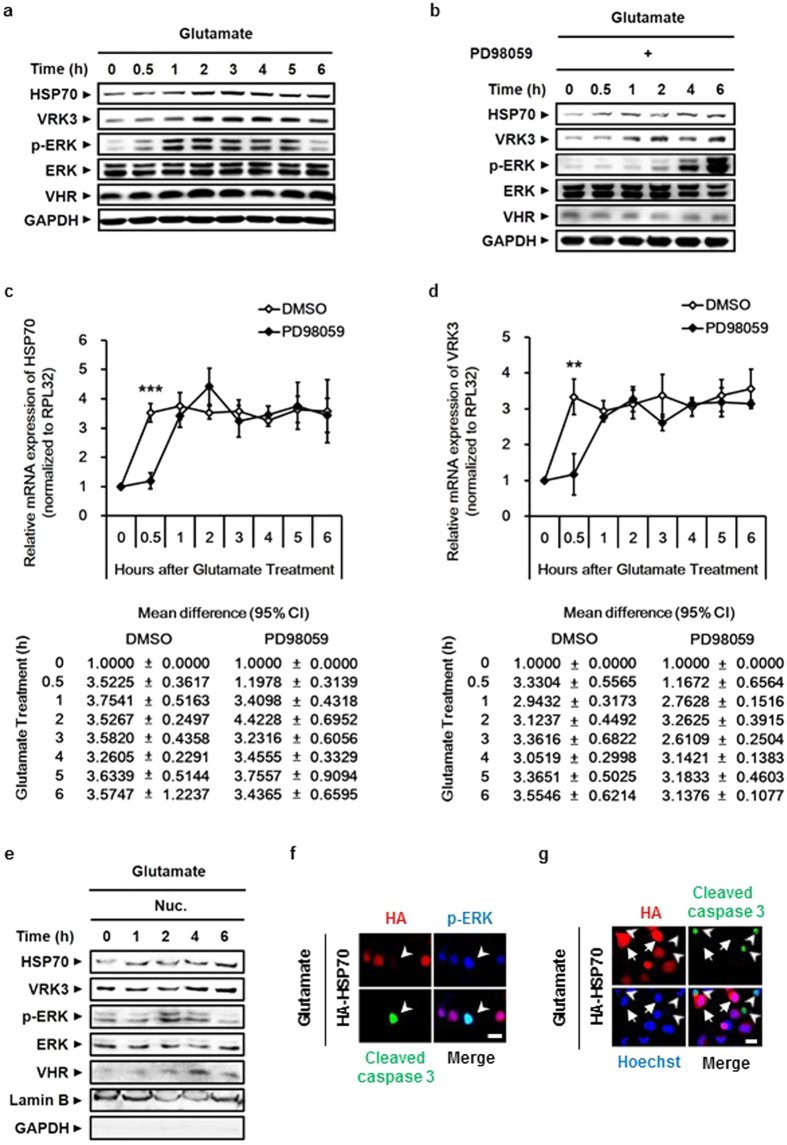
Glutamate-induced ERK activation stimulates the expression of both HSP70 and VRK3 to suppress its sustained activity that causes cell death. (**a**) The protein levels of HSP70 and VRK3 were positively correlated with ERK activity. (**b**) Blocking ERK phosphorylation using PD98059, a specific MEK/ERK inhibitor, delayed an increase in both HSP70 and VRK3 protein upon glutamate exposure. (**c**,**d**) Inhibition of ERK activation delayed mRNA expression of HSP70 (**c**) and VRK3 (**d**) following glutamate treatment. The mRNA levels of endogenous genes were detected by quantitative real-time PCR. Values are normalized to RPL32 and shown as mean ± s.d., n = 3. Student’s t-tests, **P < 0.01, ***P < 0.001. (**e**) Nuclear translocation of active ERK and increased levels of both endogenous HSP70 and VRK3 were observed in glutamate-treated SH-SY5Y cells. (**f**) HSP70-mediated downregulation of p-ERK prevented glutamate-induced apoptosis. White arrowheads highlight cleaved caspase-3-positive cells. Scale bar, 20 μm. (**g**) Glutamate-induced apoptosis was suppressed in HSP70-overexpressing SH-SY5Y cells. White arrows show HSP70-overexpressing cells. White arrowheads highlight cleaved caspase-3-positive cells. Scale bar, 20 μm.

Previous studies have shown that ERK activation induced by phorbol 12-myristate 13-acetate increases VRK3 expression^32^. Before investigating the physiological significance of both HSP70 and VRK3 in the regulation of ERK activation, we explored whether glutamate-induced ERK activity affects the expression of HSP70 and VRK3. In agreement with previous findings, treatment with glutamate stimulated ERK activation (Fig. 3a). Increased ERK phosphorylation reached a peak 1–2 h after glutamate treatment and returned to basal levels within 6 h. Moreover, we detected positive correlation between ERK activity and the protein levels of HSP70 and VRK3. To confirm an association between the activation status of ERK and the levels of HSP70 and VRK3 proteins, we pretreated SH-SY5Y cells with PD98059, a specific MEK/ERK inhibitor, before glutamate treatment (Fig. 3b). Blocking ERK phosphorylation resulted in a delayed increase in both HSP70 and VRK3 proteins upon glutamate exposure. To clarify whether the elevation of HSP70 and VRK3 protein levels followed by ERK activation is due to upregulation of endogenous mRNA levels, we performed quantitative real-time RT-PCR using SH-SY5Y cells pretreated with either vehicle or PD98059 before glutamate exposure (Fig. 3c,d). Pharmacological inhibition of ERK activation delayed mRNA expression of both HSP70 and VRK3 following glutamate treatment compared with control. After initial peak, there were no significant differences in gene expression of both HSP70 and VRK3 between the groups. These results indicate that glutamate-induced active ERK stimulates HSP70 and VRK3 expression at the transcriptional level.

Upon sustained activation, ERK 1/2 translocates to the nucleus and regulates several transcription factors, resulting in the expression of pro-apoptotic proteins^25^. Our results showed that glutamate-induced HSP70 is relocated from cytoplasm into the nucleus. To examine whether translocated HSP70 regulates nuclear ERK through the enhancement of VHR phosphatase activity, we analyzed the nucleoplasmic fraction of glutamate-treated SH-SY5Y cells (Fig. 3e). In agreement with previous findings, excessive active ERK stimulated by glutamate translocated into the nucleus, and active nuclear ERK levels were increased for up to 2 h. The levels of both endogenous HSP70 and VRK3 were also increased in the nucleus after glutamate treatment. Results from immunocytochemistry using glutamate-treated SH-SY5Y cells expressing HA-tagged HSP70 revealed that overexpression of HSP70 leads to decrease of glutamate-induced activation of ERK (Fig. 3f). Cells showed prolonged ERK activation followed by increased level of cleaved caspase 3, an active form of caspase 3 that triggers apoptosis by degrading cytoskeletal and nuclear proteins^39^,^40^. The cleavage of caspase 3 was markedly suppressed in cells overexpressing HSP70 (Fig. 3g), indicating that HSP70 inhibits neuronal cell death via prevention of glutamate-induced prolonged ERK activation in the nucleus. Taken together, these data indicate that glutamate-induced active ERK transcriptionally upregulates the expression of HSP70 and VRK3, and suppresses its prolonged activity that causes cell death.

### VRK3-mediated nuclear localization of HSP70 is important for suppression of glutamate-induced prolonged ERK activation and subsequent cell death

**Figure 4:**
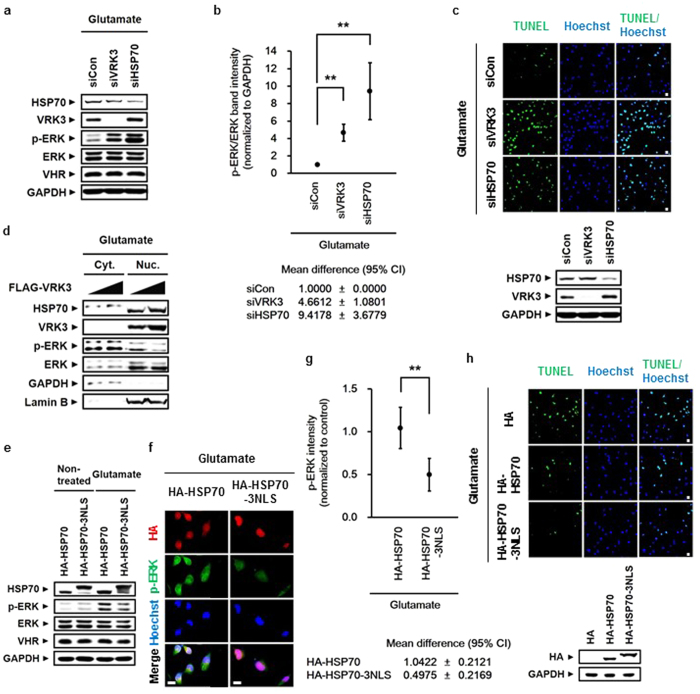
VRK3-mediated nuclear localization of HSP70 is crucial for inhibition of glutamate-induced prolonged ERK activation and subsequent cell death. (**a**) Glutamate-induced persistent ERK activation in SH-SY5Y cells after downregulation of either VRK3 or HSP70. (**b**) Quantification of the levels of p-ERK versus total ERK as in (**a**). Values are normalized to GAPDH and shown as mean ± s.d., n = 3. Student’s t-tests, **P < 0.01. (**c**) Reducing expression of either HSP70 or VRK3 rendered cells vulnerable to glutamate-induced apoptosis. Representative images of TUNEL-positive cells. Scale bar, 20 μm. (**d**) Suppression of glutamate-induced ERK activation depended on VRK3-mediated nuclear localization of HSP70. (**e**) HSP70 fused to NLS decreased glutamate-induced ERK activation more efficiently than HSP70 alone. (**f**) HSP70 nuclear localization was important for the downregulation of glutamate-induced ERK activation. Scale bar, 20 μm. (**g**) Quantification of p-ERK intensity as in (**f**). Values are normalized to control and shown as mean ± s.d., *n* ≥ 30 for each sample. Student’s t-tests, **P < 0.01. (**h**) HSP70 fused to NLS prevented glutamate-induced apoptosis more effectively that HSP70 alone. Representative images of TUNEL-positive cells. Scale bar, 20 μm.

To ascertain the involvement of HSP70 and VRK3 in reducing glutamate-induced persistent ERK activation, we examined glutamate-treated SH-SY5Y cells after downregulation of either VRK3 or HSP70 expression by siRNA (Fig. 4a–c). ERK activation in SH-SY5Y cells with either HSP70 or VRK3 knockdown was sustained for at least 6 h after glutamate exposure compared with control (Fig. 4a,b). Interestingly, sustained ERK activation led to increased expression of VRK3 in HSP70-downregulated SH-SY5Y cells, indicating the existence of a compensatory mechanism between HSP70 and VRK3 in the regulation of ERK activation. In addition, VRK3-downregulating cells which showed low levels of active ERK compared with cells with HSP70 knockdown were more susceptible to glutamate excitotoxicity-induced cell death, suggesting that VRK3 has another crucial role for survival.

We conducted TUNEL assay to verify whether the suppression of prolonged ERK activation by HSP70 and VRK3 attenuates glutamate-induced cell death. Downregulation of either HSP70 or VRK3 increased the number of TUNEL-positive cells in glutamate-treated SH-SY5Y cells (Fig. 4c). These results indicate that HSP70 and VRK3 inhibit glutamate-induced prolonged ERK activation that triggers neuronal cell death.

To confirm whether nuclear localization of HSP70 is required to decrease glutamate-induced ERK activation, we examined cytoplasmic and nucleoplasmic fractions of SH-SY5Y cells (Fig. 4d). Levels of active ERK were markedly reduced after increased nuclear localization of HSP70 along with VRK3. We created a construct capable of redirecting HSP70 to the nucleus through the fusion of three tandem repeats of NLS peptides of SV40 large T antigen (PKKKRKV)^41^ to the C-terminus of HSP70 (Fig. 4e). HSP70 fused to NLS decreased glutamate-induced ERK activation more efficiently than HSP70 alone. These results were further supported by immunofluorescence analysis (Fig. 4f,g). Notably, when HSP70 was highly concentrated in the nucleus, activate ERK in both the cytoplasm and nucleus was decreased. Moreover, TUNEL staining showed that HSP70 fused to NLS inhibits glutamate-induced apoptosis more effectively than HSP70 alone (Fig. 4h). Taken together, these results indicate that VRK3-mediated nuclear localization of HSP70 is critical for suppressing glutamate-induced persistent ERK activation and excitotoxicity-evoked neuronal cell death.

To further determine whether the interaction between the HSP70 F2 fragment and VRK3 is sufficient for HSP70 nuclear localization and subsequent downregulation of glutamate-induced ERK activation, we examined the nucleoplasmic fraction of glutamate-treated SH-SY5Y cells (Supplementary Fig. S3). Nuclear localization of HSP70 F2 fragment was increased along with VRK3. However, the F2 fragment did not have an effect on glutamate-evoked ERK activation. This observation is consistent with our finding that the HSP70 F2 fragment had almost no binding affinity towards VHR.

### Neuroprotective role of HSP70 and VRK3 against glutamate excitotoxicity in mouse cortical neurons

**Figure 5:**
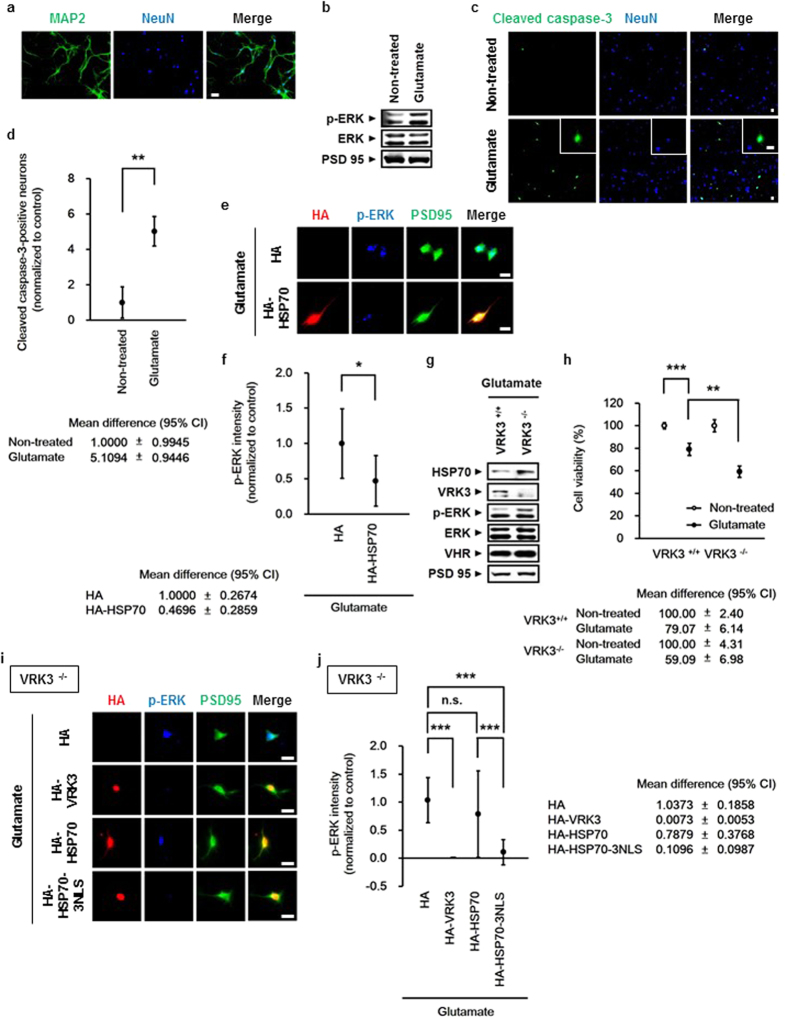
Neuroprotective role of HSP70 and VRK3 against glutamate in mouse cortical neurons. (**a**) Primary cultured mouse cortical neurons expressed MAP2 and NeuN. Scale bar, 20 μm. (**b**) Glutamate induced ERK activation in mouse cortical neurons. (**c**) Glutamate triggered apoptosis of mouse cortical neurons. Representative images of cleaved caspase-3-positive cells and NeuN-stained cell nuclei (blue). Scale bar, 20 μm. (**d**) Quantification of the percentage of cleaved caspase-3-positive neurons treated in (**c**). Values are normalized to untreated controls and shown as mean ± s.d., n = 3. Student’s t-test, **P < 0.01. (**e**) Overexpression of HSP70 downregulated glutamate-induced persistent ERK activation. Scale bar, 20 μm. (**f**) Quantification of p-ERK intensity in mouse cortical neurons expressing HA (mock) or HA-HSP70 as in (**e**). Values are normalized to HA-controls, and shown as mean ± s.d., *n* ≥ 30 for each sample. Student’s t-test, *P < 0.05. (**g**) VRK3-deficient mice exhibited excessive ERK activation induced by glutamate treatment. (**h**) VRK3-deficient mice showed increased sensitivity to glutamate-induced apoptosis. Values are normalized to untreated controls and shown as mean ± s.d., n = 3. Student’s t-test, **P < 0.01, ***P < 0.001. (**i**) Nuclear localization of HSP70 by either VRK3 or fused NLS is critical for suppressing glutamate-induced persistent ERK activation. Scale bar, 20 μm. (**j**) Quantification of p-ERK intensity in VRK3-deficient neurons expressing HA (mock), HA-VRK3, HA-HSP70, or HA-HSP70-3NLS as in (**i**). Values are normalized to HA-controls, and shown as mean ± s.d., *n* ≥ 30 for each sample. Student’s t-test, n.s., not significant, ***P < 0.001.

To determine whether HSP70 and VRK3 affect the regulation of nuclear ERK in mouse cortical neurons treated with glutamate, cortical neurons obtained at postnatal day 0.5 were purified and cultured for 5 days. Cultured cells were considered neurons based on the presence of MAP2- and NeuN-positive projections (Fig. 5a). In agreement with previous findings glutamate-evoked ERK activation was detected in mouse cortical neurons (Fig. 5b). In addition, glutamate treatment significantly increased the number of cleaved caspase-3-positive neurons (Fig. 5c,d), indicating that glutamate-induced excitotoxicity causes persistent ERK activation that triggers neuronal cell death.

Consistent with previous results, overexpression of HSP70 attenuated persistent ERK activation induced by glutamate in mouse cortical neurons (Fig. 5e,f). To directly examine the functional importance of VRK3 *in vivo,* we have used VRK3-deficient mice generated by targeted gene disruption in embryonic stem cells (Fig. 5g–j). Despite induction of HSP70, VRK3-deficient neurons showed sustained ERK activation (Fig. 5g) and markedly decreased cell viability relative to control (Fig. 5h), indicating that both HSP70 and VRK3 are essential for maintaining neuronal viability against glutamate-evoked persistent ERK activation. The importance of nuclear localization of HSP70 in the regulation of glutamate-induced ERK activation and the role of VRK3 as a facilitator of HSP70 nuclear localization were further supported by immunofluorescence analysis of VRK3-deficient neurons (Fig. 5i,j). Neurons overexpressing VRK3 exhibited markedly reduced ERK activation compared with control. Moreover, HSP70 fused to NLS decreased glutamate-induced ERK activation more efficiently than HSP70 alone, indicating that VRK3-mediated HSP70 nuclear localization and subsequent downregulation of ERK activation are required to protect neurons from glutamate-evoked excitotoxicity.

### Increased expression and nuclear localization of HSP70 suppress Aβ accumulation and neuronal cell death in brains of AD and PD patients

**Figure 6:**
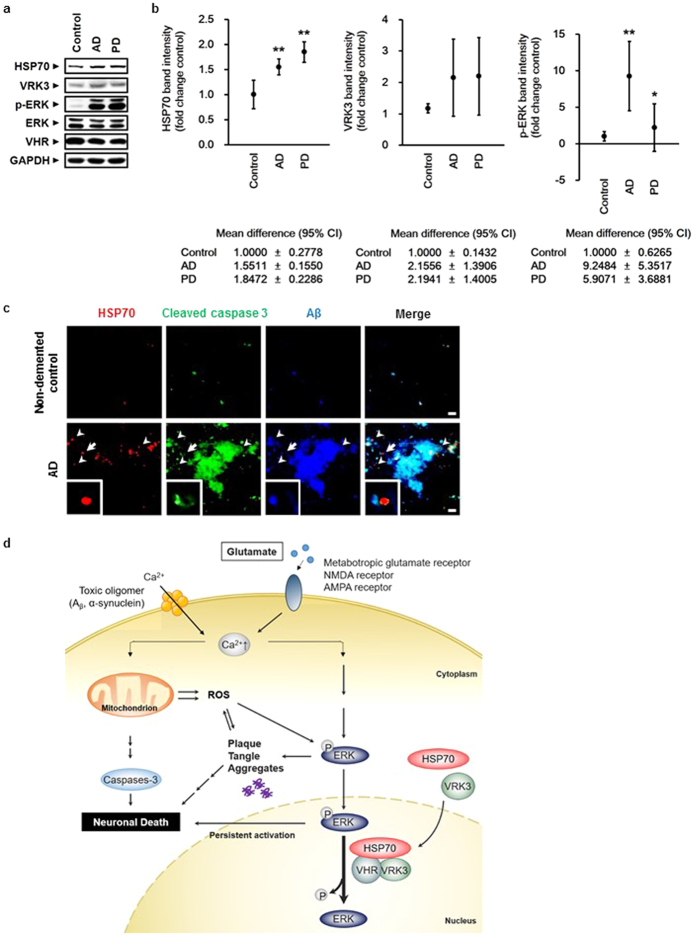
HSP70 expression is increased in brains of AD and PD patients, and especially in AD, neurons with HSP70 nuclear localization exhibit less Aβ accumulation and apoptotic cell death. (**a**) ERK activation as well as HSP70 and VRK3 expression were increased in the prefrontal cortex (PFC) of AD and PD patients compared with non-demented control individuals. (**b**) Quantification of HSP70, VRK3 and p-ERK in AD, PD, and control brains as in (**a**). Values are normalized to GAPDH and shown as mean ± s.d., n = 4. Student’s t-test, *P < 0.05, **P < 0.01. (**c**) HSP70 nuclear localization prevented Aβ aggregate formation in AD brains. White arrowheads highlight nuclear-localized HSP70. White arrows points cell enlarged. Scale bar, 10 μm. (**d**) Cytosolic calcium levels are kept low via energy-dependent sequestration into mitochondria and endoplasmic reticulum (not shown). Excessive activation of glutamate receptors and the formation of annular pores consisting of oligomeric Aβ and α-synuclein in the lipid bilayer lead to membrane permeabilization and promote excessive calcium influx. Increased intracellular calcium levels result in calcium overload of mitochondria and activation of the Ras/Raf/ERK pathway. Aβ and other amyloidogenic peptides, such as α-synuclein, generate ROS from molecular oxygen through electron transfer interactions, and impaired oxidative phosphorylation and decreased complex I activity in mitochondria also cause ROS formation ROS further impairs the ubiquitin-proteasome system, leading to accelerated accumulation of protein aggregates. In parallel, loss of mitochondrial membrane potential and persistent activation of the Ras/Raf/ERK pathway result in the release of cytochrome c from the mitochondria into the cytosol, caspase activated, and ultimately, neuronal death occurred. VRK3 facilitated nuclear localization of glutamate-induced HSP70. Nuclear HSP70 inhibited sustained ERK activation through enhancement of VHR phosphatase activity via direct binding and thereby reduced neuronal cell death.

Persistent ERK 1/2 activation and glutamate neurotoxicity are pathological features shared among neurodegenerative diseases^21^,^22^,^30^. Several studies show that HSP70 is elevated in brain tissue from elderly patients with neurodegeneration^8^,^9^. To determine whether our findings have clinical relevance, we analyzed brain lysates from AD and PD patients (Supplementary Table S1). In accordance with previous reports, HSP70 expression and p-ERK levels were significantly increased in brains of AD and PD patients (Fig. 6a,b). The mean values of VRK3 was also increased in AD and PD brains compared with normal control brains, but statistically insignificant.

Recent evidence suggests that neurodegenerative pathology is transmissible from cell to cell, and the spread of pathological proteins leads to overwhelming proteolytic stress in neurons, causing damage to specific brain regions and contributing to disease progression^42^,^43^,^44^. To determine whether VRK3-mediated HSP70 nuclear localization and subsequent downregulation of ERK activation defend against proteolytic stress by reducing aggregate toxicity, we examined prefrontal cortex sections from AD patients (Fig. 6c, Supplementary Table S2). Consistent with previous studies that showed a strong positive correlation between Aβ plaque deposition and neuronal cell death^45^, cleaved caspase-3 highly colocalized with Aβ plaque in AD brains (Fig. 6c). Interestingly, regions with high HSP70 expression exhibited less Aβ accumulation, and HSP70 predominantly localized in cell nuclei. These results indicate that increased expression of HSP70 and its nuclear localization mediated by VRK3 can contribute to neuronal protection from cell death in the brains of AD and PD patients.

## Discussion

Excitotoxicity caused by excessive activation of glutamate receptors, impairment of intracellular calcium homeostasis, increased reactive oxygen species, persistent ERK activation, and protein aggregation are implicated in neurodegenerative diseases such as AD and PD^25^,^30^,^37^,^46^,^47^,^48^. However, the endogenous protective mechanisms against neurodegeneration are poorly understood. Here, we showed that VRK3 facilitates nuclear localization of HSP70 induced by glutamate stimulation, and nuclear HSP70 enhances VHR phosphatase activity through direct binding, leading to suppression of sustained ERK activation and apoptotic cell death (Fig. 6d).

Many previous studies on the cytoprotective role of HSP70 have focused on its mediation of protein folding, degradation, and transport^5^,^49^. However, in addition to protein quality control, HSP70 may have other roles, such as the regulation of signaling pathways. Recent studies show that HSP70 modulates both caspase-dependent and -independent apoptosis by direct protein-protein interaction. For example, HSP70 interacts with and neutralizes Apoptosis-inducing factor (AIF), a mitochondrial intermembrane protein that translocates into the nucleus and triggers chromatin condensation and large-scale (~50 kbp) DNA fragmentation^50^. HSP70 can also directly bind to Apoptotic protease activation factor 1 (APAF1), which prevents APAF1 oligomerization and inhibits procaspase-9 recruitment to the apoptosome^51^,^52^. Several convincing reports illustrate the involvement of HSP70 in MAPK signaling. HSP70 suppressed ERK activation by inhibiting Mitogen-activated protein kinase kinase (MEK 1/2) activation^53^. HSP70 also inhibits RAF proto-oncogene serine/threonine-protein kinase-mediated ERK activation through competitive interactions to Bcl2-associated athanogene 1 (BAG1) that binds to and activates a RAF1 kinase^54^. The present study identified a novel regulatory mechanism of MAPK pathways by HSP70. Along with VRK3, nuclear translocated HSP70 directly binds to and enhances VHR phosphatase activity, leading to the suppression of glutamate-induced excessive ERK activation. Because HSP70 chaperone activity is regulated by ATP hydrolysis that controls substrate binding and release through conformational changes^55^, our present *in vitro* study showing HSP70-mediated enhancement of VHR phosphatase activity in the absence of ATP indicates that HSP70 facilitates VHR phosphatase activity in a chaperone activity-independent manner.

Persistent ERK activation induced by glutamate excitotoxicity and oxidative stress has been considered as one of causes to develop neurodegenerative diseases^21^,^22^,^30^. Several studies demonstrate that the upregulation of HSPs influences the progression of neurodegenerative disorders^56^,^57^ and protects against disease-associated toxicity in several neurodegenerative conditions^17^,^18^,^20^. Moreover, recently it has been reported that cyclin-dependent kinase 5-mediated phosphorylation of VRK3 at Ser 108 suppresses oxidative stress-induced prolonged ERK activation and subsequent cell death^58^. The present study investigated endogenous protective mechanisms mediated by HSP70 and VRK3 against glutamate excitotoxicity-induced neuronal cell death. Under physiological conditions, VHR is sufficient to regulate ERK activity. However, glutamate-induced excitotoxicity causes abnormal ERK activation, which impairs cellular stress resistance. Active ERK transcription upregulated both HSP70 and ERK3 expression to suppress its prolonged activity. Induced HSP70 translocates into the nucleus and enhances ERK phosphate VHR activity. VRK3 promoted HSP70 nuclear localization through binding, leading to facilitation on HSP70-mediated ERK regulation. Furthermore, increased levels of HSP70 and VRK3 observed in post-mortem brain tissue from AD and PD patients, and the nuclear localization of HSP70 is associated with less Aβ accumulation in AD brains.

These results may indicate that HSP70 and VRK3 could be targeted to reduce neurodegeneration. Both molecules have a protective role against glutamate-induced excitotoxicity in the early stages of neurodegenerative disease. Because stress-induced elevation of HSP70 does not occur across the entire brain but only in regions that are more susceptible to stress, the accumulation of severe damage in later stages of the disease may be overwhelming and cross a certain threshold that triggers cell death. These findings suggest a link between the progression of neurodegeneration and protective mechanisms against excitotoxicity-induced neuronal death. Therefore, HSP70 and VRK3 may be useful indicators for diagnosing the progressive stages of AD or PD and serve as therapeutic targets in neurodegenerative diseases.

## Methods

### Brain sample procurement

Post-mortem human brain tissue was procured in accordance with institutional guidelines and approved protocols. Formalin-fixed, paraffin-embedded prefrontal cortical brain samples and frozen prefrontal cortical brain samples from adults were obtained from the Netherlands Brain Bank. The samples analyzed in this study were strictly defined according to Braak and Braak staging^59^,^60^, with control samples being brains that were completely devoid of Lewy bodies and Aβ pathologies (i.e., Braak and Braak stage 0–2). The characteristics of these samples are provided in Supplementary Tables S1 and S2.

### Protein extraction from human brain tissue

Snap-frozen post-mortem human brain tissue was ground and homogenized in Triton lysis buffer (20 mM Tris, 150 mM NaCl, 1 mM EDTA, 1 mM EGTA, 1% Triton-X, 2.5 mM sodium pyrophosphate, 1 mM β-glycerophosphate, 1 mM Na_3_VO_4_) supplemented with protease inhibitors. Samples were sonicated and centrifuged at 15,000 rpm for 30 min at 4 °C. The supernatant was removed, and the protein concentration was determined using Bradford Reagent (AMRESCO). Samples were denatured for 5 min at 95 °C with SDS sample buffer containing β-mercaptoethanol. Proteins were separated by SDS-PAGE and transferred to nitrocellulose membranes. Western blots were quantified by densitometry using ImageJ software.

### Immunohistochemistry analysis of human brain tissue

Prefrontal cortical brain sections (4 μm thickness) were cut at regular intervals on glass slides. Paraffin-embedded tissue sections were deparaffinized in xylene and then rehydrated with decreasing concentrations of ethanol and placed in water. Sections underwent antigen retrieval using citrate buffer (10 mM sodium citrate, pH 6.0) and then were washed and blocked with PBS containing 3% bovine serum albumin and 0.2% Triton X-100 for 1 h at room temperature. Primary antibodies were diluted in blocking solution. After overnight incubation, sections were washed three times with PBS. Secondary antibodies coupled to Alexa fluorophores were diluted in blocking solution. After 1 h of incubation in the dark, sections were washed three times with PBS. For counterstaining, sections were further incubated with Hoechst (2 μg/ml) for 10 min at room temperature and then washed three times with PBS. Slices were mounted and visualized by fluorescence microscopy.

### Mice

Mice deficient for VRK3 was generated by targeted gene disruption in embryonic stem cells. Specific pathogen-free (SPF) VRK3 knockout mice were bred and maintained on a C57BL/6 background. All animals were maintained on food and water ad libitum with 12-hr light-dark cycle. Approval of the study protocol was obtained from the Pohang University of Science and Technology Institutional Animal Care and Use Committee (POSTECH IACUC). All animal experiments were carried out according to the provisions of the Animal Welfare Act, PHS Animal Welfare Policy, and the principles of the NIH Guide for the Care and Use of Laboratory Animals. All mice were maintained under conventional conditions at the POSTECH animal facility under institutional guidelines.

### Primary neuron cultures and transfection

Cortices were dissected from P0.5 newborn pups of wildtype and VRK3 knockout mice. Cultured cortical neurons were seeded onto a poly-L-lysine-coated culture dishes and were maintained in Neurobasal Medium supplemented with GlutaMAX, B27 supplements, and penicillin-streptomycin. Neuronal transfections were performed with Lipofectamine 2000 at 3 days *in vitro* (DIV) according to the manufacturer’s instructions.

### Antibodies

The following monoclonal antibodies were purchased: anti-HSP70, anti-p-ERK, and anti-α-synuclein (Abcam); anti-GAPDH (Santa Cruz Biotechnology); anti-VHR (BD Biosciences); anti-HA (Roche); anti-Aβ (BioLegend); anti-NeuN (Merck Millipore); anti-HSP70, anti-GST, anti-cleaved caspase-3, anti-Bak, and anti-Bax (Cell Signaling Technology). The following polyclonal antibodies were also obtained: anti-PSD95 (Abcam); anti-HSP70, anti-VRK3, anti-VHR, anti-p-ERK, anti-ERK, and anti-His (Cell Signaling Technology); anti-VRK3 (Atlas antibodies); anti-HA (YPYDVPDYA; Bethyl Laboratories); anti-FLAG (D-8), anti-Lamin B, and anti-MAP2 (I-18) (Santa Cruz Biotechnology).

### Plasmid construction

The coding regions of VRK3, HSP70, and VHR were amplified by RT-PCR from the mRNA of NIH3T3, SH-SY5Y, and HEK293A cells and cloned into pFLAG-CMV2, pcDNA3.1-HA, pGEX4T-1, pGEX4T-3, pEGFP-N1, or pProEX-Hta vectors. Partial fragments of HSP70 F1 (amino acids 1-380), F2 (amino acids 381-537), and F3 (amino acids 538-642) were cloned into pcDNA3.1-HA and pGEX4T-3 vectors. For nuclear expression of HSP70, three tandem repeats of NLS peptides of SV40 large T antigen (PKKKRKV)^41^ were fused to the C-terminus.

### Cell culture, transfection, and RNA interference

SH-SY5Y cells were grown in minimal essential medium (MEM) supplemented with 10% heat-inactivated fetal bovine serum (FBS) and 100 U/ml each penicillin G and streptomycin. Transient transfections were performed by electroporation using a Microporator MP-100 (Digital Bio) or Neon-Transfection System (Invitrogen). Scrambled siRNA (5′-CCU ACG CCA CCA AUU UCG U(dTdT)-3′) was purchased from Upstate Biotechnology (GE Healthcare, Dharmacon). VRK3 siRNA (5′-GGA CAA AUU GCC UUC CCA A (dTdT)-3′). HSP70 siRNA (5′-GUU UGA GGG CAU CGA CUU CU (dTdT)-3′) and VHR siRNA (5′-GGU CCU UCA UGC ACG UCA A (dTdT)-3′) were purchased from Bioneer.

### Inhibitors and glutamate treatment

The transport receptor importin-β inhibitor importazole (SML0341, Sigma-Aldrich) and the MEK/ERK inhibitor PD98059 (#9900, Cell Signaling Technology) were dissolved in dimethyl sulphoxide (DMSO) to allow addition to cell cultures at a final concentration of 0.1% (v/v) DMSO. For inhibition of importin-β, SH-SY5Y cells were pre-treated with 40 μM importazole for 1 h before glutamate treatment. For inhibition of ERK activation, SH-SY5Y cells were pre-treated with 50 μM PD98059 for 1 h. L-glutamic acid was dissolved in distilled water. SH-SY5Y cells were treated with 20 mM glutamate for 6 h. Cultures of mouse primary cortical neurons were treated with 40 μM glutamate for 24 h.

### RNA isolation and cDNA synthesis

SH-SY5Y cells were lysed with TRI-Solution (Bio Science Technology) and the total RNA was extracted after addition of 0.2 volume of chloroform. Samples were mixed with vortex, incubated at room temperature for 10 min, and centrifuged at 12,000 g for 15 min at 4 °C. Recovery of total RNA was then done by precipitation with 0.5 volume of isopropanol. After 10 min incubation at room temperature, samples were centrifuged at 12,000 g for 8 min at 4 °C. The RNA pellet was washed with 75% (v/v) ethanol, briefly air-dried, and dissolved in diethylpyrocarbonate (DEPC)-treated water by incubating for 5 min at 65 °C. Yield and purity of RNA was determined by NanoDrop 2000 spectrophotometer (Thermo Scientific). Following quantification, 1 μg of each total RNA sample was reverse transcribed using oligo-dT and the ImProm-II Reverse Transcription System (Promega) according to the manufacturer’s instructions. For qRT-PCR analysis, each cDNA sample was diluted 5 times with nuclease free water.

### Design and optimization of RT-qPCR primers

Gene-specific qPCR primers were designed using Primer3 (version 0.4.0) and Primer-BLAST (NCBI). Primer specificities were confirmed with the melting-curve after amplification by RT-qPCR. Amplicon sizes were checked by 2% agarose gel electrophoresis and ethidium bromide staining. The specificity of the primers was further confirmed by amplicon sequencing. The sequences of the forward and reverse primers were as follows: endogenous hHSP70, 5′-TGGAGTCCTACGCCTTCAAC-3′ and 5′-GATGGGGTTACACACCTGCT-3′; hVRK3, 5′-CTATTGCCCAAGTGGCAAAC-3′ and 5′-CTCAGTGTTGGGAAGGCAAT-3′; human ribosomal protein L32 (hRPL32), 5′-AACCCAGAGGCATTGACAAC-3′ and 5′-GTTGCACATCAGCAGCACTT-3′.

### Quantitative real-time PCR

The mRNA levels of endogenous genes were detected by quantitative real-time PCR using a StepOnePlus Real-Time PCR System (Applied Biosystems) with the FastStart Universal SYBR Green Master (Roche Applied Science). A 20 μl of reaction cocktail was constituted of 3 μl diluted cDNA, 10 μl 2X SYBR Green Master Mix and 0.5 μl each of the forward and reverse primers. The following amplification program was used: polymerase activation at 95 °C for 10 min; 40 repeated cycles of 95 °C for 15 s and 60 °C for 1 min. A comparative C_t_ method was used for quantification.

### Phosphatase activity assay

For measuring the *in vitro* phosphatase activity of VHR using the phosphatase substrate p-nitrophenyl phosphate (pNPP), 5 μg of GST-VHR and 10 μg of His-HSP70 were incubated in reaction buffer (50 mM Tris at pH 7.5, 5 mM MgCl_2_, 0.5 mM DTT, and 150 mM KCl) for 30 min at 30 °C. Purified proteins were further incubated with 5 × 30 mM of pNPP (P4744, Sigma-Aldrich) dissolved in recommended buffer (1 M diethanolamine at pH 10.4 with 0.5 mM MgCl_2_) for 2 h at 37 °C. The hydrolysis of the substrate was measured at 405 nm using an ELISA reader with Infinite 200 Pro NanoQuant (TECAN). For measuring the *in vitro* phosphatase activity of VHR using recombinant p-ERK2, 1 μg of GST-VHR and 2 μg of His-HSP70 were incubated in reaction buffer (50 mM Tris at pH 7.5, 5 mM MgCl_2_, 0.5 mM DTT, and 150 mM KCl) for 30 min at 30 °C. Purified proteins were further incubated with 50 ng recombinant p-ERK2 (Merck Millipore) for 1 h at 37 °C.

### Image processing

General image processing were performed by using Adobe Photoshop 7.0 (Adobe Systems Inc.).

### Statistical analysis

All of the experiments were performed at least three times. All quantitative data are presented as mean ± standard deviation (s.d.). Differences between groups were assessed using Student’s t-tests. P-values < 0.05 were considered statistically significant. *P < 0.05, **P < 0.01, ***P < 0.001.

## Additional Information

**How to cite this article**: Song, H. *et al*. VRK3-mediated nuclear localization of HSP70 prevents glutamate excitotoxicity-induced apoptosis and Aβ accumulation via enhancement of ERK phosphatase VHR activity. *Sci. Rep.* **6**, 38452; doi: 10.1038/srep38452 (2016).

**Publisher's note:** Springer Nature remains neutral with regard to jurisdictional claims in published maps and institutional affiliations.

## Supplementary Material

Supplementary Information
