# Supplementary material for: VRK3-mediated nuclear localization of HSP70 prevents glutamate excitotoxicity-induced apoptosis and Aβ accumulation via enhancement of ERK phosphatase VHR activity
Source: Sci Rep. 2016 Dec 12;6:38452. doi: 10.1038/srep38452 (PMC5150261; doi:10.1038/srep38452)
Supplement: Supplementary Information [file srep38452-s1.pdf]

VRK3-mediated nuclear localization of HSP70 prevents glutamate excitotoxicity-induced apoptosis and Aβ accumulation via enhancement of ERK phosphatase VHR activity

Haengjin Song[1], Wanil Kim[1], Sung-Hoon Kim[2], and Kyong-Tai Kim[1, 2, *]

[1]Department of Life Sciences, Pohang University of Science and Technology, Pohang, Gyeongbuk, 37673, Republic of Korea. [2]Division of Integrative Biosciences and Biotechnology, Pohang University of Science and Technology, Pohang, Gyeongbuk, 37673, Republic of Korea.

[*] Correspondence and requests for materials should be addressed to K.-T.K (email: ktk@postech.ac.kr)

SUPPLEMENTARY DATA

Supplementary Methods

Supplementary Figures

- **Supplementary Fig. S1** Overexpression of FLAG-tagged VRK3 didn't affect the nuclear localization of HSP70 in SH-SY5Y cells under the normal conditions.
- **Supplementary Fig. S2** Neither overexpression nor knockdown of VHR influences nuclear localization of HSP70 in glutamate-treated SH-SY5Y cells.
- **Supplementary Fig. S3** The HSP70 F2 fragment containing the SBD was not sufficient to decrease glutamate-induced ERK activation.

Supplementary Tables

- **Supplementary Table. S1** Clinicopathological details of subjects used for Western blotting
- **Supplementary Table. S2** Clinicopathological details of subjects used for immunohistochemistry

Supplementary Methods

Purification of fusion proteins. All GST- or His-tagged fusion proteins were expressed in Escherichia coli BL21 (DE3) pLysS (Novagen) and purified using glutathione-sepharose 4B agarose beads (GE Healthcare Bio-Sciences) or Ni-NTA agarose (Invitrogen) according to the manufacturer's instructions.

In vitro binding assay. For GST-pulldown assay, 3 µg of GST-tagged full length HSP70 and its fragments were incubated overnight with 2 µg of VHR in Triton lysis buffer containing 1 mM DTT and 10% glycerol, followed by incubation with glutathione-sepharose 4B beads (GE Healthcare Bio-Sciences) at 4 °C for 1 h. GST was used as negative control.

Immunoprecipitation. Cells were washed with chilled phosphate-buffered saline (PBS) and lysed for 30 min on ice using Triton lysis buffer supplemented with protease inhibitors (Roche). Lysates were sonicated and clarified by centrifugation at 15,000 rpm for 30 min. Immunoprecipitation was performed by incubating antibodies with lysates overnight followed by incubation with protein G beads (Roche) at 4 °C for 1 h.

Preparation of cytoplasmic and nuclear extracts. To prepare cytoplasmic extracts, SH-SY5Y cells were lysed at 4 °C in a lysis/extraction buffer containing 10 mM HEPES (pH 7.6), 3 mM $MgCl_2$, 40 mM KCl, 0.5% NP-40, 5% glycerol, 2 mM DTT, and 0.5 mM PMSF supplemented with protease inhibitors. After incubation on ice for 15 min, cellular debris was removed by centrifugation (3,000 rpm) at 4 °C for 5 min. Nuclei were resuspended and spun down twice in lysis/extraction buffer to avoid contamination of cytoplasmic proteins. To prepare nuclear extracts, pelleted nuclei were placed in nuclear extraction buffer containing 10 mM HEPES (pH 7.9), 0.1 mM EGTA, 1.5 mM $MgCl_2$, 420 mM NaCl, 25% glycerol, 0.5 mM DTT, and 0.5 mM PMSF. After incubation on ice for 15 min, samples were sonicated and centrifuged (3,000 rpm) at 4 °C for 5 min. The supernatant was removed, and protein concentration was analyzed using Bradford reagent (AMRESCO).

Assessment of cell viability and apoptosis. Cell viability was assessed using a chromogenic assay involving biological reduction of the tetrazolium salt 3-(4,5-dimethylthiazol-2-yl)-2,5-diphenyltetrazoliumbromide (MTT), which is converted into blue formazan crystals by living cells. After electroporation with DNA or siRNA, cells were subcultured in 96-well plates at a density of 1×10^4 cells for 24 h in a final volume of 100 µl. Cells were then cultured in serum-free medium supplemented with glutamate for the indicated period of time. After the medium was removed, MTT (5 mg/ml in PBS) was added to the culture medium. Cells were incubated for 2 h in the dark at 37 °C, and the cell-free supernatant was removed from the wells. A total of MTT solvent (4 mM HCl and 0.1% Nondet P-40 [NP40], both in isopropanol) were added to dissolve the formazan crystal, and absorbance was measured at 570 nm using an ELISA reader with Infinite 200 Pro NanoQuant (TECAN). Apoptotic cells were further visualized using a modified TdT-mediated dUTP Nick End Labeling (TUNEL) assay with a DeadEnd Fluorometric TUNEL system (Promega) according to the manufacturer's protocol.

Immunofluorescence. Transfected cells were grown on 0.1% gelatin-coated glass chip in 24-well plates. Cells were fixed in 4% paraformaldehyde 24 h after initial plating, permeabilized with 2% NP-40, then blocked and incubated overnight with 10% FBS in PBS containing primary antibodies. Primary antibodies were visualized with Alexa-350 (anti-mouse, Life Technologies), Alexa-488 (anti-rabbit, anti-mouse, and anti-goat, Invitrogen), and Alexa-594 (anti-rabbit and anti-rat, Invitrogen) secondary antibodies. Nuclei were visualized with 2 µg/ml Hoechst. Slides were mounted with Dako Fluorescent Mounting Medium (Dako) and visualized by fluorescence microscopy (Axioplan2, Zeiss; Olympus IX71, Olympus).

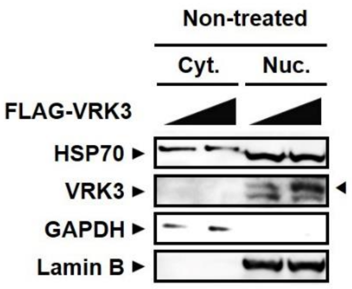

Supplementary Fig. S1. Overexpression of FLAG-tagged VRK3 didn't affect the nuclear localization of HSP70 in SH-SY5Y cells under the normal conditions. Nuclear localization of the HSP70 was not altered in SH-SY5Y cells overexpressing FLAG-tagged VRK3.

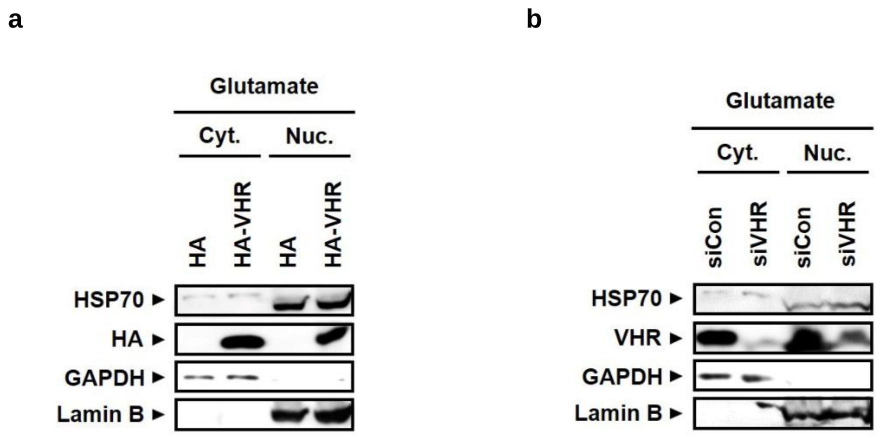

Supplementary Fig. S2. Neither overexpression nor knockdown of VHR influences nuclear localization of HSP70 in glutamate-treated SH-SY5Y cells. (**a**) Nuclear localization of the HSP70 was not different between control and VHR overexpressing cells. (**b**) Reducing expression of VHR using siRNAs did not affect the level of HSP70 in the nucleus.

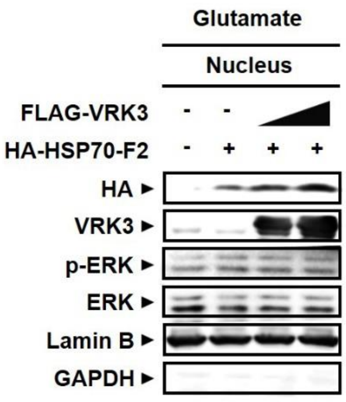

Supplementary Fig. S3. The HSP70 F2 fragment containing the SBD was not sufficient to decrease glutamate-induced ERK activation. Nuclear localization of the HSP70 F2 fragment lacking VHR binding affinity was not sufficient to downregulate glutamate-evoked ERK activation.

Supplementary Table S1. Clinicopathological details of subjects used for Western blotting.

Profile and Clinical data of AD and PD patients and control cases for Western blotting

Diagnosis	NBB no	Age(yr)	Sex	Braak	Amyloid	Braaklb	PMD(hours)
Control	12-070	79	M	2			05:45
	09-300	71	V	1	A		07:10
	12-049	70	V	2	A		07:35
	12-059	78	V	2	A		04:35
AD	08-107	77	M	4	C		04:05
	09-185	70	M	4	C		04:00
	12-022	79	M	4	C		04:05
	07-315	71	M	5	B		05:25
PD	11-117	78	M			4	06:15
	04-108	73	M	1	A	5	05:35
	09-207	67	V	1	B	6	07:40
	09-235	70	M	1	B	5	05:15

NBB no, Netherlands Brain Bank number; Braak: braak stage based on amyloid-beta(0-2: non-demented AD, 3-6: mild to severe)

Amyloid, a type of amyloid; Braaklb, braak stage based on lewy body (0-2: non-demented PD, 3-6: mild to severe);

PMD, post-moterm delay; Control, non-demented control; AD, Alzheimer's disease; PD, Parkinson's disease; M, male.

Supplementary Table S2. Clinicopathological details of subjects used for immunohistochemistry.

Profile and Clinical data of AD and PD patients and control cases for immunohistochemistry

Diagnosis	NBB no	Age(yr)	Sex	Braak	Amyloid	Braaklb	PMD(hours)
Control	11-091	76	M	0	O	0	06:45
	05-074	79	M	1	A	1	06:30
	12-070	79	M	2			05:45
	12-104	79	M	2	A		06:30
	09-300	71	V	1	A		07:10
AD	08-107	77	M	4	C		04:05
	09-185	70	M	4	C		04:00
	12-022	79	M	4	C		04:05
	07-315	71	M	5	B		05:25
	11-002	71	V	5	C		04:15
	05-154	67	V	6	C		06:05

NBB no, Netherlands Brain Bank number; Braak: braak stage based on amyloid-beta(0-2: non-demented AD, 3-6: mild to severe)
Amyloid, a type of amyloid; Braaklb, braak stage based on lewy body (0-2: non-demented PD, 3-6: mild to severe);
PMD, post-moterm delay; Control, non-demented control; AD, Alzheimer's disease; PD, Parkinson's disease; M, male.
